# Developing a CoMSIA Model for Inhibition of COX-2 by Resveratrol Derivatives

**Published:** 2016

**Authors:** Jamal Shamsara, Ahmad Shahir-Sadr

**Affiliations:** a *Pharmaceutical Research Center, School of Pharmacy, Mashhad University of Medical Sciences, Mashhad, Iran. *; b *Bioinformatics Research Center, Sabzevar University of Medical Sciences, School of Medicine, Sabzevar, Iran.*

**Keywords:** 3D-QSAR, COX-2, COMSIA, Inhibitors, Resveratrol, Selectivity

## Abstract

Design of selective cyclooxygenase-2 (COX-2) inhibitors is still a challenging task because of active site similarities between COX isoenzymes. To help with this issue, we tried to generate a 3D-QSAR (3 dimensional quantitative structure activity relationships) model that might reflect the essential features of COX-2 active sites. Compounds in a series of resveratrol derivatives inhibitors with reported biological activity against COX-2 were used to construct a predictive comparative molecular similarity indices (CoMSIA) model. A CoMSIA model with acceptable internal and external predictability was developed and employed to design new not yet synthesized molecules with improved activity and selectivity toward COX-2. Finally, molecular docking of the inhibitors in COX-2 active site demonstrated the possible ability of proposed compounds to inhibit COX-2, selectively.

## Introduction

Plants, in particular grapes, nuts and berries, are the main sources for resveratrol (3,5,4′-trihydroxystilbene) that is a natural polyphenolic compound. *Vitis vinifera*, *labrusca* and *muscadine* are three types of grapes contain maximum concentration of resveratrol. During the past years several beneficiary effects for the resveratrol were proposed. It has shown the ability to slow down the progression of various conditions, including cancers, cardiovascular diseases, and ischemic injuries, as well as enhance stress resistance and extend lifespan (1, 2). However, the experimental basis for such health benefit is not fully understood. One of the suggested mechanisms is its anti-inflammatory properties. It exerts the anti-inflammatory effect through different pathways. The inhibition of enzymes involved in the inflammatory response, such as cyclooxygenase-1 (COX-1) or cyclooxygenase-2 (COX-2) is one of the main targets for resveratrol (3).

COX, a key enzyme in prostaglandin (PG) synthesis, has two isoforms, COX-1 and COX-2. COX-1 is constitutively expressed in most cells, whereas COX-2 is induced by inflammatory stimuli suggesting that COX-2 plays a critical role in inflammation (4). COX-2 also plays key roles in generation of tumor (5-7). Multiple direct targets of resveratrol have been identified. Studies have shown the ability of resveratrol and its derivatives in direct inhibition of COX-2 enzyme (8, 9). 

In this study a CoMSIA study on a series of resveratrol derivative COX-2 inhibitors was carried out. Comparative molecular similarity indices (CoMSIA) is a method for 3D-QSAR (3 dimensional quantitative structure activity relationship) studies that its reliability has been established (10). 

## Experimental

*Methods*

*Obtaining biological data and generation of molecular structures*

**Figure 1:**
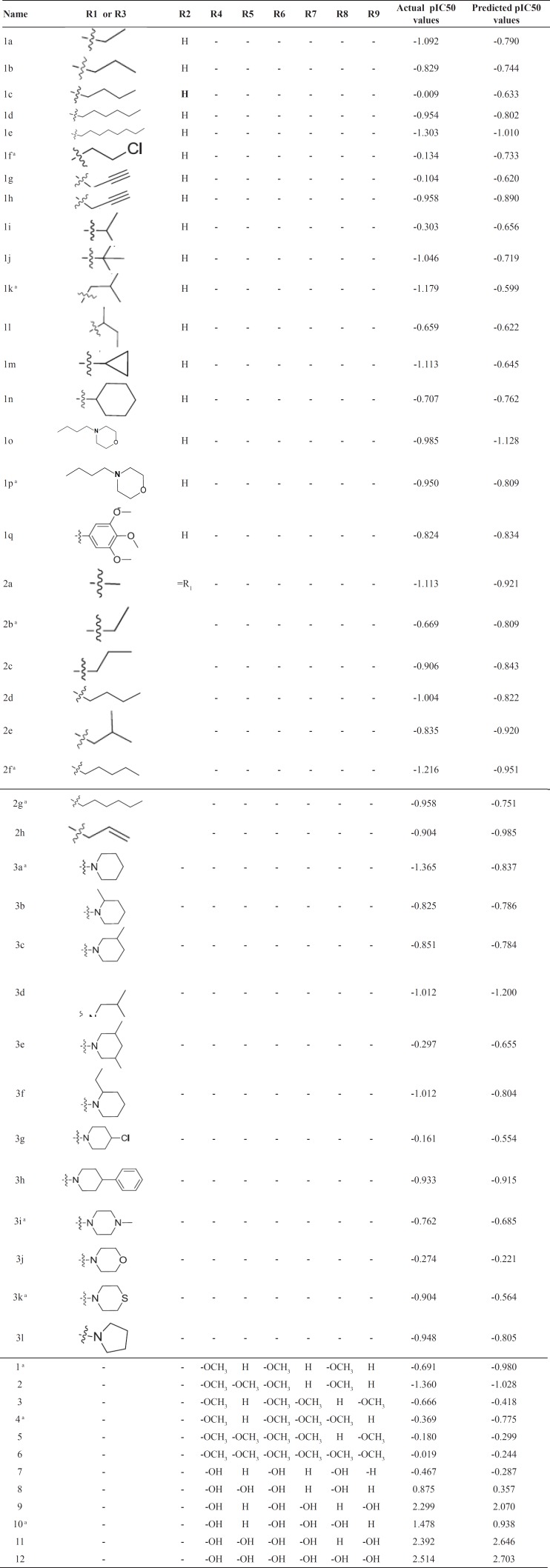
General structures for data set. (A) For structures 1a-1n and 2a-2h. (B) For structures 3a-3l. (C) For structures 1-12

The structure of 49 resveratrol derivatives and their biological activities for inhibitors of COX-2 were taken from the literatures (11, 12) (Figure 1. and Table 1.). Celecoxib was used as a control in both studies. We normalized the IC50 based on reported activity for celecoxib. The range of pIC50 (µM) values for COX-2 spans at least three orders of magnitude (min = -1.365, max = 2.514) in training set. The compounds were divided into two sets, Training (n = 37) and test (n = 12) sets according to the maintaining of structural diversity and the uniformly distribution of IC50. The pIC50 (-Log IC50) was employed as dependent variable instead of IC50.

The molecular structures were built using PyMOL (www.pymol.org, The PyMOL Molecular Graphics System, Version 1.2r3pre, Schrödinger, LLC.). The Gasteiger-Huckel partial charges for all compounds were assigned and then, 3D conformation of the compounds was minimized using the standard Tripos force field (Tripos International, St. Louis). The CoMSIA model was developed by SYBYL-X1.2 molecular modeling package (Tripos International, St. Louis).

*Alignment*

The alignment of 3D molecular structures is a crucial step to have a reliable CoMSIA model. An ideal alignment is a one that can result in superimposed similar functional groups of different ligands onto each other and mimic the active conformations of ligands during the interaction with the receptor. In the CoMSIA study on COX-2 inhibitors we have used rigid structure alignment using Distill module (in SYBYL-X1.2 molecular modeling environment). Compound 1c was selected as template for Distill alignment. This compound was chosen for its potent COX-2 inhibition property. 

*CoMSIA model generation and validation*

CoMSIA (like CoMFA) similar to other 3D-QSAR methodologies tries to correlate 3D conformation of the ligands with their biological activity. The default CoMSIA setting was used in this study. For developing a CoMSIA model, firstly, a large grid box was positioned around the aligned compounds with default spacing value of 2 Å. Then, the default probe (a sp3 carbon atom with 1.0 Å van der Waals radius and +1 net charge) experienced different interaction energies for each individual molecule at every grid point. Five descriptors (strict, electrostatic, hydrophobic, H-bond donor and acceptor fields) were evaluated using the probe. These fields value form a large table that might have correlation with experimental and biological values (in this case, pIC50).

After generation of descriptors, Partial least square (PLS) regression was used to find the possible correlation between dependent variable (-pIC50) and independent variable (CoMSIA generated descriptors). At this step, q^2^ and standard error of prediction (SEP) obtained from leave-one-out cross validation roughly estimate the predictive ability of the model. This cross validated analysis followed by a non-cross validated analysis with the calculated optimum number of principle components. Conventional correlation coefficient r^2^, standard error of estimate (SEE) and F value indicated the validity of the model. Finally, a set of compounds (which were not present in model development process) with observed activity were used for external validation of the generated model. Predictive r^2^ (r^2^_pred_) value was calculated using:

r^2^_pred _= 1 – PRESS/SD

PRESS: sum of the squared deviation between predicted and actual pIC50 for the test set compounds

SD: sum of the squared deviation between the actual pIC50 values of the compounds from the test set and the mean pIC50 value of the training set compounds. 

The output of the CoMSIA model can be viewed as a contour map that graphically shows the favorable and unfavorable positions for specific interaction fields around the aligned molecules using polyhedrons in different colors. We set the favored and disfavored levels to default values of 80% and 20%, respectively. 

*Prediction set (design of new compounds)*

The prediction set contained 11 new not yet synthesized compounds having unknown observed values of activity against COX-2 (Table 2.) They were designed based on the developed CoMSIA model.

*Molecular Docking*

The molecular docking process was carried out employing Glide (Glide, version 5.7, Schrödinger, LLC, New York, NY, 2011) using default parameters. The protein (1CX2) was prepared using Protein Preparation Wizard. Hydrogens were added, bond orders were assigned, overlapping 

hydrogens were corrected, missing side chains were added and water molecules were removed. Finally the protein structure was minimized by OPLS2005 force field. The prepared protein structure containing inhibitor molecule was used for active site definition (within 13 Å from co-crystalized ligand). The 2D maps of ligands-receptor interactions were generated by ligand interaction diagram. 

**Table 1:** Actual and predicted activities of the training and test sets according to the CoMSIA model. Activities were shown as pIC50 (µM

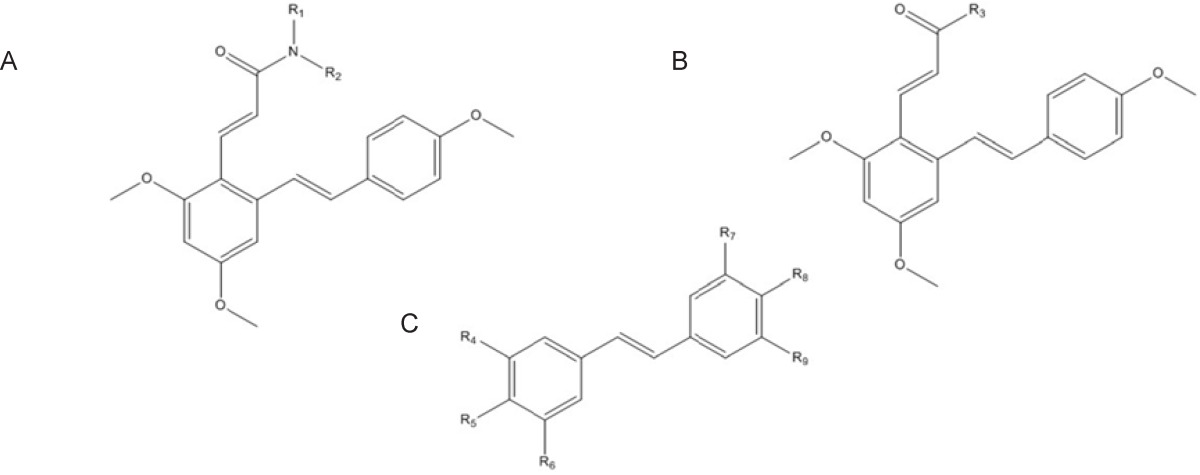

a : test set compounds.

**Table 2:** Actual and predicted activities of the training and test sets according to the CoMSIA model. Activities were shown as pIC50 (µM

**Name**	**Predicted pIC50 values**	**Docking score**
n1	-0.643	-4.38668
n2	-0.502	-7.70702
n3	-0.565	-5.16976
n4	3.019	-10.7421
n5	3.008	-11.5014
n6	3.046	-9.72465
n7	2.98	-11.3252
n8	3.359	-7.96709
n9	3.255	-10.2527
n10	3.406	-7.23175
n11	3.368	-8.44834
1c (observed= -0.009)	-0.633	-7.49537
12 (observed= 2.514)	2.703	-9.3481

**Table 3:** Statistical characteristics of the developed CoMSIA model

**Parameter**	**Value**
Number of compounds included in training set	37
LOO q^2^	0.787
SEP	0.474
Optimum number of principal components	4
r^2^	0.925
SEE	0.280
F values	99.140
Steric field %	0.055
Electrostatic field %	0.232
Hydrophobic	0.123
Hydrogen Donor	0.210
Hydrogen acceptor	0.379
r^2^_pred_	0.733

**Figure 2:**
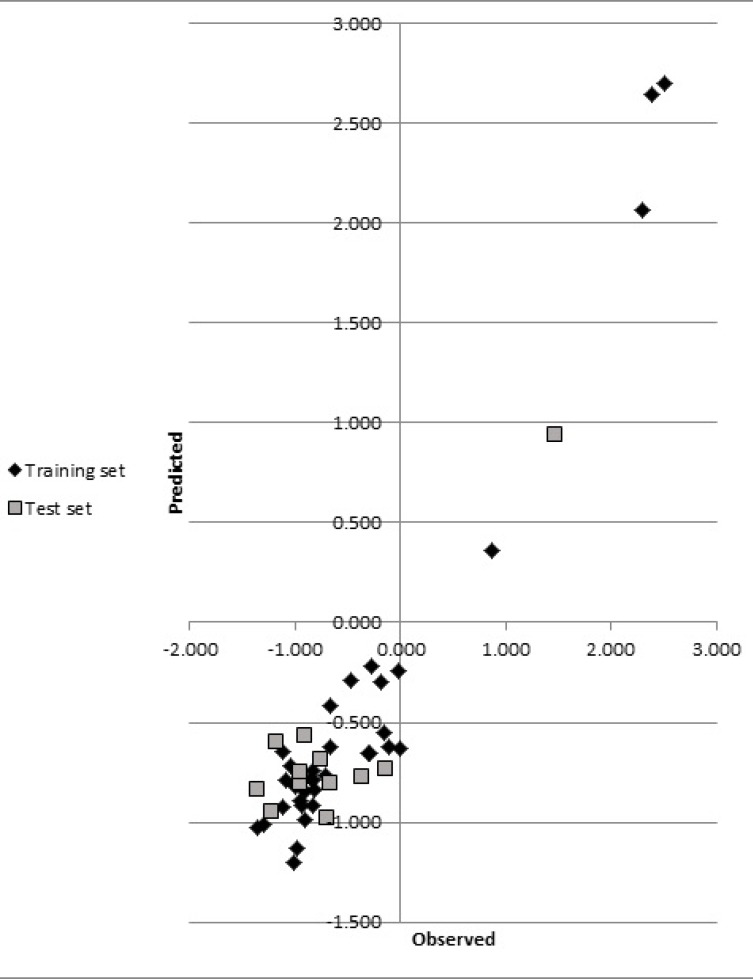
Plots of the predicted against observed activity for training and test sets

**Figure 3:**
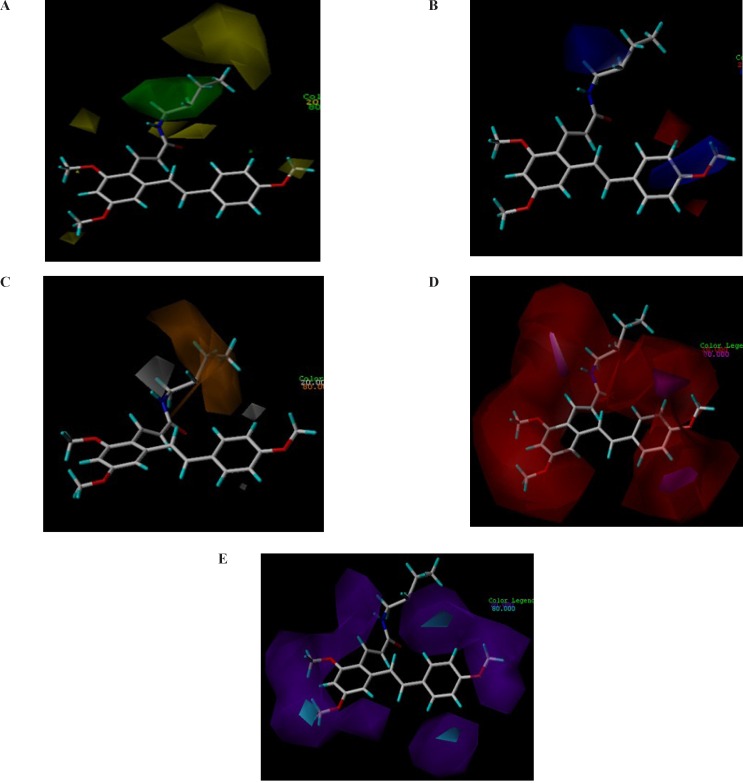
CoMSIA contour maps. Steric and electrostatic contours for COX-2 are presented. (A) Green and yellow contours show regions of steric tolerance and intolerance, respectively. (B) Red and blue contours show regions where negative and positive electrostatic potential, respectively, are favored. (C) Hydrophobic contours for COX-2 are also illustrated. The orange contours are favored while gray contours are disfavored for hydrophobic interactions. Hydrogen bond donor-acceptor contours for COX-2 are shown in (D) and (E). The regions enclosed by magenta polyhedron are favored for hydrogen acceptors while disfavored ones are enclosed by red polyhedron (D). The cyan contours are favored regions for hydrogen donors while the purple polyhedrons are disfavored for them (E

**Figure 4:**
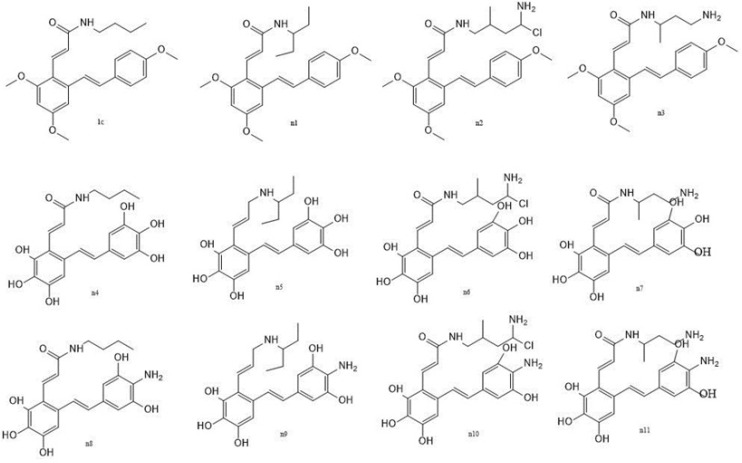
Chemical structures of new but not yet synthesized molecules

**Figure 5:**
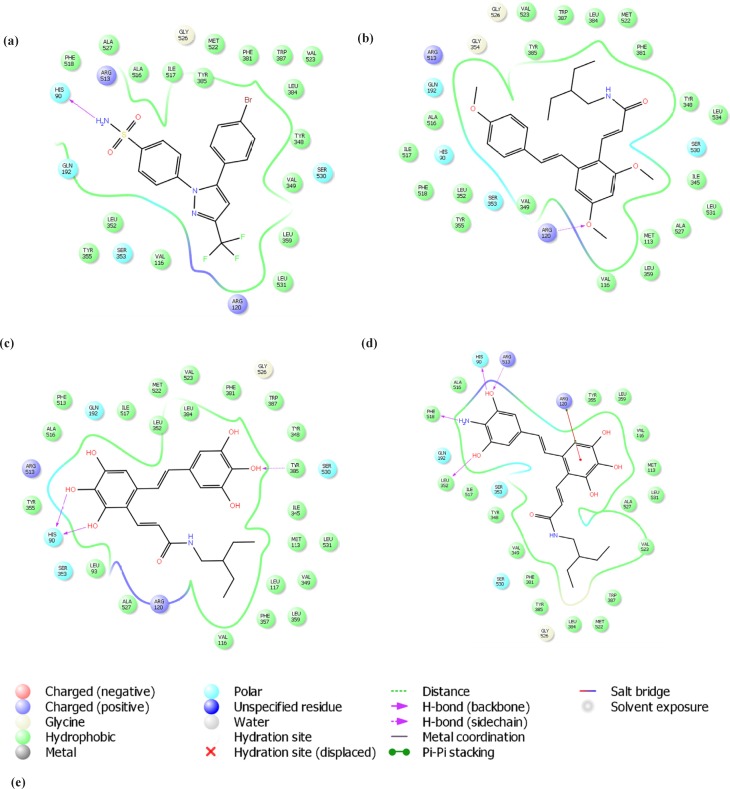
2D interaction diagram of (A) co-crystallized ligand of 1CX2 PDB complex and 3 docked designed compounds (B) n1 (C) n5 and (D) n9 with COX-2 receptor. Legend was presented (E

## Results

*CoMSIA predictivity*

The statistics for developed CoMSIA model were shown in Table 3. The statistical parameters, q^2^, SEP, r^2^, F, SEE and r^2^_pred_ showed the validity of our model. The predicted biological activities were shown in Table 1. The results of r^2^_pred_ calculation showed that the proposed CoMSIA model was reliable and could successfully predict pIC50 for structurally related compounds which were not included in development of the models. Predicted pIC50 for training and test set were presented in Table 1. The experimental pIC50 against the values predicted by the CoMSIA models were plotted (Figure 2).

*CoMSIA contour analysis*

The contour maps of the CoMSIA model have summarized the favored and disfavored 3D structural features of ligands–COX-2 interactions which are responsible for ligands activity. We investigated the generated CoMSIA models to find out key futures of activity. 

 The contour maps of the CoMSIA steric, electrostatic, hydrophobic, hydrogen bond acceptor and hydrogen bond donor fields are shown in Figure 3.

*Prediction set (design of new virtual compounds)*

This work allowed the prediction the activity of a set shown in Figure 4., not yet synthesized molecules. Their inhibitory activities were calculated according to the CoMSIA model. They were designed based on compound 1c. As compound 1c is the most active compound among structures 1a-3l (scaffold a or b), we tried to use the information derived from the developed QSAR model to improve the activity of compound 1c. We have proposed a library of 11 new structures, some of them may exhibit great COX-2 inhibitory activity (Table 2.) compared to the parent compound. This hypothesis should be verified experimentally.

We modified the aliphatic chain of 1c according to the steric contour map (Figure 3a.). The positive and other polar groups were introduced to this position to satisfy the electrostatic and hydrophobic field contours (Figure 3b and 3c). The methyl groups at R4 – R9 groups were omitted based on the hydrophobic disfavored regions shown in 3a by yellow polyhedrons and hydrogen bond donor and acceptor favored regions shown by magenta and purple contours in Fig. 3d and 3e, respectively. 

*Molecular docking*

The molecular docking approach was employed to further analysis the ability of designed compounds in inhibition of COX-2. In Table 2. the docking scores of the 11 new designed compounds and 2 templates were reported. For some of them the 2D diagram of ligand-receptor interaction was presented (Figure 5b-d). The binding positions of all new compounds were inspected for their binding conformation and interaction with COX-2 active site.

## Discussion

We successfully developed a CoMSIA model for prediction of some resveratrol derivatives inhibition activity against COX-2. Subsequently, the model was used to predict new resveratrol amides COX-2 inhibitor activity. The improved potency of new not yet synthesized molecules further was evaluated by molecular docking. 

COX isoenzymes (COX-1 and COX-2) have similar active sites located at the end of a long and narrow hydrophobic pocket. However, slight changes in amino acid composition of the hydrophobic channel make the shape of COX-2 active site different. A substitution of Ile523 in COX-1 by Val523 residue in COX-2 increases accessibility to inner parts of the COX-2 active site. In other words, the COX-2 substrate channel is wider, due to the presence of the smaller Valine residue. Thus, three hydrophilic residues Phe518, Arg513 and Hist90 petitioned in the side pocket of COX-2 are able to form hydrogen bonds with hydrophilic groups (13, 14). The COX-2-specific inhibitors celecoxib, rofecoxib and valdecoxib have a diarylheterocyclic structure, whereas nonselective NSAIDs have carboxylic or enolic acid group to bind to the Arg120 residue in COX-1. COX-2-specific inhibitors has a bulky side chain to discourage binding in the narrower hydrophobic channel of the COX-1 and a hydrophilic side chain to encourage binding in the hydrophilic side pocket of the COX-2 (15). In Figure 5a a COX-2-specific inhibitor (SC-558) was shown inside the active site of the COX-2 enzyme (PDB code: 1CX2). The smaller Valine residue at position 523 makes the hydrophilic side pocket accessible more than one of COX-1. Within this pocket, the Arg513, Phe518 and His90 residues can form hydrogen bonds with the side chains on selective inhibitors results in blocking access of the natural substrate, arachidonic acid, to the catalytic site at Tyr385.

As it was shown earlier the methylated resveratrol derivatives have lower binding affinity for COX enzymes than the resveratrol its own and its hydroxylated derivatives (11). Our CoMSIA model quantified this and it was shown graphically in steric contour map of the model. Docked structures in the active site of COX-2 also indicated that none-methylated derivatives make hydrogen bonds Specially with Arg120, Ser530, and Tyr385, which is in accordance to the results observed with other none-selective NSAIDs while the methylated derivatives lake such strong hydrogen binding network. As previously reported these hydroxylated derivatives (molecules 7-12) could not able to have access to the additional side pocket (that is only accessible in COX-2 due to substitution of a Val523 in COX-2 for an isoleucine in the active site of COX-1) reported to be responsible for COX-2 selectivity. 

In the next part of the study increase in COX-2 affinity of resveratrol amide derivatives were investigated. The most potent and selective resveratrol amide derivative reported by the author (12) was 1c. We tried to make small changes in the side chain of this compound according to the obtained CoMSIA model to improve potency. Virtually designed compound n1 was docked in COX-2 active site. The side chain rested in selectivity side pocket of COX-2 active site but it was still have no significant hydrogen bond interaction with active site residues. The calculated docking score was also low. To have more potent selective COX-2 inhibitors we designed none-methylated resveratrol amide derivatives. Structures n5 and n9 had good predicted activities predicted by the obtained CoMSIA model and also docking scores. Furthermore they showed the ability to interact with specific residues in side pocket of COX-2 active site. It was shown that selective COX-2 inhibitors side chains form hydrogen bonds with Phe518, Arg513, and His90 residues. The co-crystallized ligand (SC-558) which is a selective COX-2 inhibitor forms hydrogen bonds with His 90 and Arg513 (Figure 5a). Compounds n5 and n9 also formed hydrogen bond with His 90. Furthermore they interacted effectively with other residues at COX-2 active site. In SC-558 three fluorine are positioned near the positively charged residue Arg120. In n5, the carboxyl group was placed near that residue. NH_2_ group in n9 compound make an extra interaction with Phe518. In addition, they have no hydrogen bond interaction with Arg120 which is a characteristic for none selective inhibitors.

In summary, we have developed a reliable CoMSIA model for resveratrol-derived COX-2 inhibitors using activity data as reported (11, 12). We used CoMSIA analyses, to design new not yet synthesized potent and selective COX-2 inhibitors. Their potency and selectivity were confirmed by docking studies and should also be investigated experimentally.
